# Orthonormal pairwise logratio selection (OPALS) algorithm for compositional data analysis in high dimensions

**DOI:** 10.1093/bioadv/vbaf229

**Published:** 2025-10-01

**Authors:** Paulína Jašková, Javier Palarea-Albaladejo, Karel Hron, Dominik Lachman, Matthias Templ, Magali Berland

**Affiliations:** Department of mathematical analysis and applications of mathematics, Faculty of Science, Palacký University Olomouc, Olomouc 77146, Czech Republic; Institute of Active Lifestyle, Faculty of Physical Culture, Palacký University Olomouc, Olomouc 77111, Czech Republic; Department of Computer Science, Applied Mathematics and Statistics, University of Girona, Girona 17003, Spain; Department of mathematical analysis and applications of mathematics, Faculty of Science, Palacký University Olomouc, Olomouc 77146, Czech Republic; Department of Algebra and Geometry, Faculty of Science, Palacký University Olomouc, Olomouc, 77146, Czech Republic; School of Business, FHNW University of Applied Sciences and Arts Northwestern Switzerland, Olten 4600, Switzerland; Université Paris-Saclay, INRAE, Jouy-en-Josa MGP 78350, France

## Abstract

**Summary:**

In the analysis of compositional data, the most fundamental information is conveyed by the pairwise logratios between components. While logratio coordinate representations, such as balances and pivot coordinates, are widely used to aggregate such information into higher-level relationships, there are instances where a fine-grained representation using all pairwise logratios can be advantageous. Performing this within an orthonormal (or orthogonal) logratio coordinate framework becomes particularly challenging for high-dimensional compositions, since a composition with *D* parts results in D(D−1)/2 pairwise logratios (excluding reciprocals). This work presents an efficient algorithm (OPALS) based on Latin squares theory to obtain all orthonormal pairwise logratios from just D−1 logratio coordinate systems. Thus, the computational burden associated with using such representation for data analysis and modelling in high dimensions is notably alleviated, or even made feasible. Moreover, the relationship between estimates from orthonormal pairwise logratios and ordinary pivot coordinates is discussed in the context of regression and classification analysis.

**Availability and implementation:**

The OPALS algorithm is described in detail in this article and can be implemented directly from the provided methodology. The performance and properties of the method are illustrated through two examples using contemporary molecular biology data.

## 1 Introduction

Compositional data analysis (CoDA) is a well-established statistical methodology for the analysis of multivariate data representing parts of a whole. Pairwise logratios (PLRs hereinafter) are the most fundamental representation of compositional data and the starting point for their proper statistical analysis. They carry the essential relative information as scale invariant objects (Pawlowsky-Glahn *et al.* 2015). Since the seminal monograph Aitchison (1986), developments in CoDA have led to the introduction of more elaborated logratio representations, e.g. the popular balances (Egozcue and Pawlowsky-Glahn 2005) and pivot coordinate (Filzmoser *et al.* 2018) representations. These aim to somehow aggregate such information so that major relevant relationships are succinctly captured. They are specific instances of the class of so-called orthonormal logratio (olr) coordinates (Martín-Fernández 2019, Egozcue *et al.* 2003), which are well rooted into the algebraic-geometrical structure of the sample space of compositions (Pawlowsky-Glahn *et al.* 2015). However, particularly in the high-dimensional context, such representations can lead to logratio coordinates involving large collections of compositional parts, which might be related in complex and heterogeneous ways, with the values of the coordinates eventually resulting from some mixed processes. This can challenge interpretability and the objective of disentangling the most basic underlying processes.

Resorting to work directly with the elemental PLRs can be a sensible way to overcome these difficulties in some applications. However, there are issues with arbitrary choices of PLR representations and their compatibility with the formal structure of the sample space of compositions as pointed out in Hron *et al.* (2021) and Nesrstová *et al.* (2023a). We argue that these are even more relevant in the high-dimensional context in relation to common regression and classification problems, often involving questions related to dimension reduction, variable selection, computational efficiency and stability, etc. Building on Hron *et al.* (2021) and Nesrstová *et al.* (2023a), where so-called backwards pivot coordinates (bpcs) were introduced as a consistent formulation of PLRs within an orthonormal or orthogonal logratio coordinate framework, we here extend such idea to the case of high-dimensional compositions. In brief, each bpc system contains one PLR, which is accompanied by other more complex logratios to complete a full orthonormal coordinate system. Although the entire system consisting of D−1 coordinates is used in the analysis, the focus is just on results involving such PLR coordinate of interest. Eventually, D(D−1)/2 bpcs (each one extracted from the corresponding orthonormal system) are required to represent a *D*-part composition.

However, extending such an approach to the high-dimensional case is confronted with some new challenges. Thus, while the default bpcs are workable when the numbers of compositional parts is relatively small, as soon as these increase the number of coordinate systems involved increases quadratically. For instance, working with just 10 parts already requires 45 backwards coordinate systems. And, considering e.g. studies in the omics sciences where dealing with hundreds of parts is ordinary, having say 450 parts would lead to the astonishing figure of 101 025 coordinate systems being required. Consequently, a computationally efficient method is a must in this context so that orthonormal coordinate systems in the style of bpcs can be devised. Particularly, being able to incorporate as many PLRs as possible per system, instead of just one, would be a notable improvement. Note also that sticking to orthonormal coordinate systems is desirable [or even just orthogonal, which enhances the interpretability of coordinates (Müller *et al.* 2018)]. Namely, in the regression context, this guarantees that the regression coefficients of PLR coordinates remain the same irrespectively of the coordinate system they are extracted from.

Given the above, a combinatorial procedure drawing from Latin squares theory (Casselgren and Häggkvist 2013) is proposed, which guarantees that each orthonormal coordinate system contains D/2 non-overlapping PLRs. The remaining D/2−1 coordinates are irrelevant for our purpose, but are nevertheless required in order to complete each orthonormal system. These are then obtained by applying the ordinary sequential binary partition (SBP) scheme (Egozcue and Pawlowsky-Glahn 2005). Using such strategy, called the Orthonormal Pairwise Logratio Selection (OPALS) algorithm, only D−1 coordinate systems are actually needed to gather the information about all D(D−1)/2 PLRs and, hence, the computational burden is significantly reduced. Thus, this work contributes methodological grounds to work efficiently with PLRs in high-dimensional problems. Moreover, their relationship with ordinary pivot coordinates is stressed in the context of regression and classification analysis. In our view, considering all PLRs along with such aggregated forms can enrich the analysis and provide more detailed insight into the nature of the associations and patterns in the data.

In the following, Section 2 presents bpcs as a special case of the more general concept of balance coordinates. Section 3 introduces the novel OPALS algorithm to obtain orthonormal PLRs following the bpc approach in a computationally efficient way. Section 4 presents two illustrative examples using high-throughput molecular biology data sets. Lastly, Section 5 concludes with some final remarks and future outlook.

## 2 Orthonormal coordinate representations for compositional data

The logratio methodology for the statistical analysis of compositional data involves mapping from their original space of scale invariant objects into the real space. A number of logratio representations have been proposed for this and it is common in CoDA that different ones are used for different purposes. Thus, additive logratio (alr) coordinates (Aitchison 1982) have been traditionally used to represent PLRs. The set of alr coordinates are obtained by choosing one part as divisor part and then dividing all the others by it before taking logs. Formally, for a *D*-part composition $x=(x_{1},\ldots ,x_{D})$ and divisor $x_{D}$, alr coordinates are defined as


(1) $$\text{alr}(x)=\left(\ln\frac{x_{1}}{x_{D}},\ldots ,\ln\frac{x_{D-1}}{x_{D}}\right).$$

However, although simple and useful for different purposes, alr coordinates present the general issue that they result from an oblique coordinate system and are not compatible with the algebraic-geometrical structure of compositional data, so-called Aitchison geometry (Pawlowsky-Glahn *et al.* 2015), consisting of an Euclidean geometry. In this case, using alr coordinates can cause problems in combination with techniques involving Euclidean distances and makes them inappropriate. Or they lead to inconsistencies and associated interpretability issues in a regression context (McGregor *et al.* 2020, Coenders and Pawlowsky-Glahn 2020, Hron *et al.* 2021).

Alternatively, so-called pivot coordinates are derived from an orthonormal coordinate system and aim to capture all the relative information about a certain part in one logratio coordinate (Filzmoser *et al.* 2018). They are formally defined as


(2) $$z_{i}=\sqrt{\frac{D-i}{D-i+1}}\ln\frac{x_{i}}{\sqrt[{D-i}]{\prod_{d=i+1}^{D}x_{d}}},\;i=1,\ldots ,D-1.$$

Note that the first pivot coordinate $z_{1}$ involves the logratio of the part $x_{1}$ to the geometric mean of all the other parts. Thus, rearranging x so that the *l*th part is placed at the first position, i.e. $x^{\left[l\right]}=\left(x_{1}^{\left[l\right]},\ldots ,x_{D}^{\left[l\right]}\right)=(x_{l},x_{1},\ldots ,x_{l-1},x_{l+1},\ldots ,x_{D})$ with  l=1,…,D, the associated pivot coordinates $z^{\left[l\right]}=\left(z_{1}^{\left[l\right]},\ldots ,z_{D-1}^{\left[l\right]}\right)$ sequentially isolate the role of the *l*th component with respect to the others in the first coordinate, l=1,…,D:


(3) $$\begin{array}{c} z_{1}^{\left[l\right]}=\sqrt{\frac{D-1}{D}}\;\ln\frac{x_{l}}{\sqrt[{D-1}]{\prod_{\overset{d=1}{d\neq l}}^{D}x_{d}}}=\frac{1}{\sqrt{D(D-1)}}\cdot \\ \cdot (\ln\frac{x_{l}}{x_{1}}+\cdots +\ln\frac{x_{l}}{x_{l-1}}+\ln\frac{x_{l}}{x_{l+1}}+\cdots +\ln\frac{x_{l}}{x_{D}}). \end{array}$$

In this manner, the first pivot coordinate summarizes all the relative information about $x_{l}$ via the aggregation of all the PLRs with $x_{l}$ in the numerator. It is important to note that all the *D* pivot coordinate systems are just orthogonal rotations of each other. Also note that the set of first pivot coordinates is closely related to another common logratio representation, centred logratio (clr) coefficients $c=(c_{1},\ldots ,c_{D})$ (Aitchison 1986):


(4) $$c_{l}=\ln\frac{x_{l}}{\sqrt[{D}]{\prod_{d=1}^{D}x_{d}}}=\sqrt{\frac{D-1}{D}}z_{1}^{\left[l\right]},\;l=1,\ldots ,D.$$

This relationship is particularly useful in high-dimensional settings to reduce computational burden, since it allows replacing dealings involving *D* pivot coordinate systems by straightforward manipulation of clr coefficients while keeping (up to scaling) the same interpretation.

Furthermore, pivot coordinates are just a special case of the family of olr coordinates known as balances (Egozcue and Pawlowsky-Glahn 2005). Balances result from a SBP of a *D*-part composition x into non-overlapping subsets of parts. Accordingly, the balance at the *k*th partition is given by


(5) $$b_{k}=\sqrt{\frac{r_{k}s_{k}}{r_{k}+s_{k}}}\;\ln\frac{{\left(x_{i_{1}}x_{i_{2}}\ldots x_{i_{r_{k}}}\right)}^{1/r_{k}}}{{\left(x_{j_{1}}x_{j_{2}}\ldots x_{j_{s_{k}}}\right)}^{1/s_{k}}},\;k=1,\ldots ,D-1,$$

where indices $i_{1},i_{2},\ldots ,i_{r_{k}}$ denote the $r_{k}$ parts of the first subset (coded by 1 in the table representing the SBP and going into the numerator of the logratio) and $j_{1},j_{2},\ldots ,j_{s_{k}}$ denote the $s_{k}$ parts of the second subset (coded by −1 in the SBP table and going into the denominator). At any step, a part not involved in the balance is coded by 0. For illustration, Table 1 shows the particular SBP table leading to pivot coordinates (2).

**Table 1.** Sequential binary partition of a composition leading to ordinary pivot logratio coordinate representation.

	$x_{1}$	$x_{2}$	$x_{3}$	$x_{4}$	⋯	$x_{D-1}$	$x_{D}$
$z_{1}$	1	−1	−1	−1	⋯	−1	−1
$z_{2}$	0	1	−1	−1	⋯	−1	−1
$z_{3}$	0	0	1	−1	⋯	−1	−1
⋯	⋮	⋮	⋮	⋮	⋯	⋮	⋮
$z_{D-1}$	0	0	0	0	⋯	1	−1

With the aim to provide an alternative to alr coordinates (1) to represent PLRs within an orthonormal coordinate framework, Hron *et al.* (2021) introduced the concept of bpcs. Following on the idea of ordinary pivot coordinates, the purpose is to devise olr coordinate systems so that any PLRs of interest is represented by the first coordinate, with the remaining coordinates of each system obtained by SBP, but somehow in the reverse direction. Following Nesrstová *et al.* (2023a), D−1 bpc systems can be obtained as


(6) $$\text{bpc}{\left(x^{\left(l\right)}\right)}_{i}=\sqrt{\frac{i}{i+1}}\;\ln\frac{x_{i+1}^{\left(l\right)}}{\sqrt[{i}]{\prod_{j=1}^{i}x_{j}^{\left(l\right)}}},\;i=1,\ldots ,D-1,$$

where $x^{\left(l\right)}=(x_{1}^{\left(l\right)},\ldots ,x_{D}^{\left(l\right)})$, l∈{1,…,D−1}, stands for the permutation of the parts in x so that the *l*th part is placed at the second position and the divisor part (e.g. $x_{D}$ for the sake of simplicity) is placed at the first position. That is, $x^{\left(l\right)}=(x_{D},x_{l},x_{1},\ldots ,x_{l-1},x_{l+1},\ldots ,x_{D-1})$ (note that the reordering of parts is for convenience not the same as in $x^{\left[l\right]}$ above, and we use parenthesis instead of brackets for the superscript to emphasize this). Then the PLR of interest (the pivoting coordinate) is given by


(7) $$\text{bpc}{\left(x^{\left(l\right)}\right)}_{1}=\frac{1}{\sqrt{2}}\;\ln\frac{x_{2}^{\left(l\right)}}{x_{1}^{\left(l\right)}}=\frac{1}{\sqrt{2}}\;\ln\frac{x_{l}}{x_{D}}.$$

The SBP table for the particular case of l=1 is depicted in Table 2. For later use in Section 3.3, we also detail here the case where a general divisor $x_{r}$, with r≠l, is considered; and denote the resulting bpcs by $\text{bpc}{\left(x^{\left(lr\right)}\right)}_{i}$ for i=1,…,D−1 and r=1,…,D, with $x^{\left(l\right)}\equiv x^{\left(lD\right)}$. By considering all (l,r) combinations with l>r (i.e. up to reciprocals), the resulting D(D−1)/2 bpc systems are obtained.

**Table 2.** Sequential binary partition of a composition leading to backwards pivot coordinates for l=1.

	$x_{1}$	$x_{2}$	$x_{3}$	$x_{4}$	⋯	$x_{D-1}$	$x_{D}$
$\text{bpc}{\left(x^{\left(1\right)}\right)}_{1}$	1	0	0	0	⋯	0	−1
$\text{bpc}{\left(x^{\left(1\right)}\right)}_{2}$	−1	1	0	0	⋯	0	−1
$\text{bpc}{\left(x^{\left(1\right)}\right)}_{3}$	−1	−1	1	0	⋯	0	−1
⋯	⋮	⋮	⋮	⋮	⋯	⋮	⋮
$\text{bpc}{\left(x^{\left(1\right)}\right)}_{D-1}$	−1	−1	−1	−1	⋯	−1	1

Finally, note that the requirement of orthonormality could be relaxed in regression analysis by replacing it by just orthogonality (Müller *et al.* 2018), which in practice implies removing the normalizing constants in (6). This contributes to simplifying the interpretation of the regression coefficients. Therefore, orthogonal counterparts to balance-like coordinates will be used below for convenience.

## 3 Efficient orthonormal coordinate representation of pairwise logratios

Following on the previous section, D(D−1)/2 bpc systems are required in order to generate all possible PLRs and carry out the corresponding statistical processing on them. However, this procedure becomes practically unfeasible in the context of high-dimensional compositional data due to its excessive computational burden, particularly where other computationally intensive tasks such as cross-validation or bootstrapping might be also involved. To overcome this, we here outline an efficient approach to construct olr coordinate systems that, instead of just one, contain as many PLRs as possible in each of them. In particular, D−1 coordinate systems including D/2 non-overlapping PLRs each suffice to obtain all the D(D−1)/2 PLRs required. The procedure draws on the theory of Latin squares introduced by Casselgren and Häggkvist (2013).

### 3.1 Latin squares

Our combinatorial problem of selecting D/2 non-overlapping PLRs within D−1 olr coordinate systems such that each PLR is contained exactly once has a precise translation into the language of Latin squares theory. Specifically, this involves finding a symmetric Latin square with a constant diagonal, a problem that has multiple solutions as described in Casselgren and Häggkvist (2013). A Latin square of order *D* is a D×D array *L* of elements from a set {1,…,D} such that each element of the set {1,…,D} occurs in each row and in each column exactly once. Being *L* a Latin square of order *D*, this will be symmetric if for each i,j≤D, it happens that L(i,j)=L(j,i). Moreover, *L* will be diagonal if all the elements on the first diagonal are equal.

Let *I* be a set with *D* elements, I={1,…,D}, where *D* is even (otherwise we assume that omitting the odd element will not affect the analysis, which is definitely a reasonable assumption in a high-dimensional context). Let *X* be the set of unordered pairs of elements of *I*, i.e. *X* has D(D−1)/2 elements, and let consider the subsets $X_{1},\ldots ,X_{D-1}\subseteq X$ satisfying the following properties:

1.
$X=X_{1}\cup \cdots \cup X_{D-1}$ .
2. The sets $X_{i}$ are pairwise disjoint, i=1,…,D−1.
3. For each k=1,…,D−1, the set $X_{k}$ contains pairwise disjoint elements. That is, for each couple of unordered pairs $\{\{a,b\},\{c,d\}\}\in X_{k}$, either {a,b}={c,d} or {a,b}∩{c,d}=∅.

The following theorem shows that such partition of *X* corresponds to a certain symmetric Latin square with constant diagonal and rows and columns indexed by *I*. Such Latin square is further analysed in Casselgren and Häggkvist (2013).

Theorem 1. *Let L be a* D×D  *array of elements from the set* {1,…,D}  *with diagonal entries all equal to D. Being* I={1,…,D}  *and X the set of unordered pairs of elements of I, let consider subsets* $X_{k}\subseteq X$*, for* k=1,…,D−1*, such that*
 

(8) $$\{i,j\}\in X_{k}\Leftrightarrow L(i,j)=k=L(j,i).$$

*Then* $X_{1},\ldots ,X_{D-1}$  *satisfy properties (i)–(iii) if and only if L is a symmetric diagonal Latin square with diagonal entries equal to D.*

**Proof.** Assuming *L* to be a symmetric Latin square with diagonal constantly equal to *D* implies that there are only elements 1,…,D−1 beyond such diagonal. Consequently, each {i,j}∈X belongs to a unique subset $X_{1},\ldots ,X_{D-1}$, which yields (i)–(ii). To verify (iii), let assume for some $X_{k}$ that there are pairs $\{\{i,l\},\{j,l\}\}\in X_{k}$ with i=j. Then, in the *l*th column of *L*, the element *k* would occur at least twice (in the *i*th and *j*th rows), which would contradict that *L* is a Latin square. Next, let assume that $X_{1},\ldots ,X_{D-1}$ defined by (8) satisfy properties (i)–(iii). We first show that *L* is a symmetric array. By property (ii), any {i,j}∈X belongs to some $X_{k}$, so that L(i,j)=k=L(j,i), which confers symmetry. Under the assumption that *L* is diagonal, the following proves that *L* is a Latin square. Given the symmetry, it is enough to prove that each row of *L* contains each element of k∈{1,…,D} at most once. The fact that *D* occurs only in the diagonal confirms this for the k=D case. For k=D, if there were indexes l,i,j∈I with i=j such that L(l,i)=k=L(l,j), then it would obviously happen that i,j=l and, hence, having $\{\{l,i\},\{l,j\}\}\in X_{k}$ would contradict property (iii).

### 3.2 Orthonormal pairwise logratio selection (OPALS) algorithm

The concept of Latin squares drafted above is applied here to devise a procedure to optimally extract all PLRs from a given composition. As stated above, this involves defining D−1 orthonormal coordinate sets, each including D/2 unique and non-overlapping PLRs, that jointly lead to the D(D−1)/2 PLRs sought after.

Following on the previous section, let $I_{2},\ldots ,I_{D}$ be a collection of D−1 sets, each representing pairs of integers {i,j} with i,j≤D. For an even *k*, the *k*th set is given by


(9) $$\begin{array}{c} I_{k}=\{\{i,j\}|i+j=k+1\}\\ \;\cup \{\{i,j\}|i,j\neq D,\;i+j=D+k\}\\ \;\cup \{\{D,(D+k)/2\}|D\neq k\}, \end{array}$$

whereas for an odd *k* it is


(10) $$\begin{array}{c} I_{k}=\{\{i,j\}|i+j=k+1\}\\ \;\cup \{\{i,j\}|i,j\neq D,\;i+j=D+k\}\\ \;\cup \{\{D,(k+1)/2\}|D\neq k\}. \end{array}$$

For our purposes, the pairs {i,j} in each system $I_{k}$ are the indexes of the compositional parts used to form the PLRs (note that setting i<j leads to the required D(D−1)/2 PLRs in total). Given $I_{k}=\{I_{k,1},\ldots ,I_{k,D/2}\}$, an olr system can be defined by considering the corresponding D/2 PLRs along with any other compatible olr coordinates obtained by SBP to complete the system. Any SBP can be used so that, after the first D/2−1 steps, it leads to D/2 two-part logratios formed by elements with indices in $I_{k}$. For example, without loss of generality, given $I_{k}=\{\{1,2\},\{3,4\},\ldots ,\{D-1,D\}\}$, the balances


$$b_{j}^{\left(k\right)}=\sqrt{\frac{2D-4j}{D-2j+2}}\ln\frac{{\left(x_{2j-1}x_{2j}\right)}^{1/2}}{{\left(\prod_{m=2j+1}^{D}x_{m}\right)}^{D-2j}},$$

for j=1,…,D/2−1, along with


$$b_{D/2}^{\left(k\right)}=\frac{1}{\sqrt{2}}\ln\frac{x_{1}}{x_{2}},\ldots ,b_{D-1}^{\left(k\right)}=\frac{1}{\sqrt{2}}\ln\frac{x_{D-1}}{x_{D}},$$

form the required olr coordinate system. Table 3 depicts the corresponding SBP.

**Table 3.** Sequential binary partition to obtain complete orthonormal logratio coordinate system including selected pairwise logratios.

	$x_{1}$	$x_{2}$	$x_{3}$	$x_{4}$	$x_{5}$	$x_{6}$	⋯	$x_{D-3}$	$x_{D-2}$	$x_{D-1}$	$x_{D}$
$b_{1}^{\left(k\right)}$	1	1	−1	−1	−1	−1	⋯	−1	−1	−1	−1
$b_{2}^{\left(k\right)}$	0	0	1	1	−1	−1	⋯	−1	−1	−1	−1
⋯	⋮	⋮	⋮	⋮	⋮	⋮	⋯	⋮	⋮	⋮	⋮
$b_{D/2-1}^{\left(k\right)}$	0	0	0	0	0	0	⋯	1	1	−1	−1
$b_{D/2}^{\left(k\right)}$	1	−1	0	0	0	0	⋯	0	0	0	0
$b_{D/2+1}^{\left(k\right)}$	0	0	1	−1	0	0	⋯	0	0	0	0
⋯	⋮	⋮	⋮	⋮	⋮	⋮	⋯	⋮	⋮	⋮	⋮
$b_{D-1}^{\left(k\right)}$	0	0	0	0	0	0	⋯	0	0	1	−1

The structure of the OPALS algorithm is summarized in Algorithm 1. To have an empirical assessment of its computational performance, we measured running time and random access memory (RAM) space usage when applied to compositional data with increasing dimensionality *D*. The results summarized in Table 4 show that the elapsed time grows cubically, while the peak memory usage grows quadratically with *D*. For example, considering a composition of D=300 parts, the procedure completes the task in ∼14 minutes, reaching a peak memory usage of 597 MB. And for D=450, the execution requires nearly 51 min and 1313 MB of RAM. This involves the cost of constructing the D−1 orthonormal coordinate systems including D/2 non-overlapping PLRs. Formally, we can set the asymptotic time and space complexities to be about $O(D^{3})$ and $O(D^{2})$ respectively. These bounds align with Table 4, reflecting the scalability of the OPALS algorithm, which remains computationally feasible for moderate to high-dimensional settings.

**Table 4.** Runtime and memory usage of the OPALS algorithm for increasing number *D* of compositional parts.

	*D*								
	50	100	150	200	250	300	350	400	450
Elapsed time (min)	0.07	0.52	1.8	4.1	8.2	14.5	23	34.8	50.9
Peak RAM used (MB)	58.7	100.5	197.5	283.1	408.1	596.9	885.6	1026.4	1313.4

Algorithm 1 OPALS

**Input:** Composition $x=(x_{1},\ldots ,x_{D})$ with $x_{i}>0$ for all *i*

**Output:**  D−1 orthonormal coordinate systems $I^{\left(2\right)},\ldots ,I^{\left(D\right)}$, each containing D−1 balances, together representing all D(D−1)/2 pairwise logratios

**Initialization:** Let k=2,…,D denotes index of the coordinate system.

1. **Construct index sets**  $I_{k}$**:** 
  - For even *k*:
    Ik={{i,j}|i+j=k+1}∪{{i,j}|i,j=D,i+j=D+k}∪{{D,(D+k)/2}|D≠k}
  - For odd *k*:
    Ik={{i,j}|i+j=k+1}∪{{i,j}|i,j=D,i+j=D+k}∪{{D,(k+1)/2}|D≠k}
  Each $I_{k}$ contains D/2 disjoint index pairs $\left\{i_{m},j_{m}\right\}$ with $i_{m}<j_{m}$ and m=1,…,D/2.
2. **Construct coordinate system**  $I^{\left(k\right)}$  **using index set**  $I_{k}$**:** 
  - For each pair $\{x_{i_{m}},x_{j_{m}}\}\in I_{k}$, m=1,…,D/2−1 construct a balance coordinate $b_{m}^{\left(k\right)}$ using the SBP (the corresponding row in the SBP from Table 3, for the special case elaborated there):
    $$b_{m}^{\left(k\right)}=\sqrt{\frac{2D-4m}{D-2m+2}}\ln\frac{{\left(x_{i_{m}}x_{j_{m}}\right)}^{1/2}}{{\left(\prod_{p=m+1}^{D}x_{i_{p}}x_{j_{p}}\right)}^{D-2m}},$$
  along with
$$b_{D/2}^{\left(k\right)}=\frac{1}{\sqrt{2}}\ln\frac{x_{i_{1}}}{x_{j_{1}}},\ldots ,b_{D-1}^{\left(k\right)}=\frac{1}{\sqrt{2}}\ln\frac{x_{i_{D/2}}}{x_{j_{D/2}}},$$
**Repeat for all**  k=2,…,D  **to obtain all coordinate systems.** 

**Final Output:**  $\overset{D-1}{\underset{k=1}{\cup }}I^{\left(k\right)}$ gives a complete set of orthonormal coordinate systems containing all pairwise logratios.

#### 3.2.1 Illustration for a composition of *D* = 6 parts

Let consider a 6-part composition $x=(x_{1},\ldots ,x_{6})$. Hence, five sets $I_{2},\ldots ,I_{6}$ will suffice to obtain all 6·×·5/2=15 PLRs following the proposed algorithm. Thus, for k=2, we have that


$$\begin{array}{c} I_{2}=\{\{i,j\}|i+j=3\}\cup \{\{i,j\}|i,j\neq 6\wedge i+j=8\}\\ \cup \{\{6,8/2\}\}=\{\{1,2\},\{3,5\},\{4,6\}\}. \end{array}$$

Simplifying the expressions by omitting the normalization constants (i.e. considering orthogonal and not orthonormal coordinates), this set gives rise to the PLRs $\left\{\ln\frac{x_{1}}{x_{2}},\ln\frac{x_{3}}{x_{5}},\ln\frac{x_{4}}{x_{6}}\right\}$. The remaining four sets and associated PLRs are generated analogously:

-
I3={{i,j}|i+j=4}∪{{i,j}|i,j=6  &  i+j=9}∪{{6,4/2}}={{1,3},{4,5},{2,6}}⇒{lnx1x3,lnx4x5,lnx2x6} ,
-
$I_{4}=\{\{1,4\},\{2,3\},\{5,6\}\}\Rightarrow \{\ln\frac{x_{1}}{x_{4}},\ln\frac{x_{2}}{x_{3}},\ln\frac{x_{5}}{x_{6}}\}$ ,
-
$I_{5}=\{\{1,5\},\{2,4\},\{3,6\}\}\Rightarrow \{\ln\frac{x_{1}}{x_{5}},\ln\frac{x_{2}}{x_{4}},\ln\frac{x_{3}}{x_{6}}\}$ ,
-
$I_{6}=\{\{1,6\},\{2,5\},\{3,4\}\}\Rightarrow \{\ln\frac{x_{1}}{x_{6}},\ln\frac{x_{2}}{x_{5}},\ln\frac{x_{3}}{x_{4}}\}$ .

For each $I_{k}$, the corresponding olr system including the PLRs is completed by adding compatible balances by SBP. Different SBPs could be used for this purpose [including e.g. the one used to construct pivot coordinates (2)]. It is important to note though that the results of any subsequent statistical analysis will not depend on this choice. For instance, Table 5 depicts a possible SBP associated to $I_{2}$ which generates the D−1=5 olr coordinates (up to normalizing constants) given by


$$I^{\left(2\right)}=(\ln\frac{{\left(x_{1}x_{2}x_{3}x_{5}\right)}^{1/4}}{{\left(x_{4}x_{6}\right)}^{1/2}},\ln\frac{{\left(x_{1}x_{2}\right)}^{1/2}}{{\left(x_{3}x_{5}\right)}^{1/2}},\ln\frac{x_{1}}{x_{2}},\ln\frac{x_{3}}{x_{5}},\ln\frac{x_{4}}{x_{6}}).$$

**Table 5.** Illustrative sequential binary partition table associated to first system $I_{2}$ from the OPALS algorithm for a 6-part composition.

Order	$x_{1}$	$x_{2}$	$x_{3}$	$x_{4}$	$x_{5}$	$x_{6}$	*r*	*s*
1	1	1	1	−1	1	−1	4	2
2	1	1	−1	0	−1	0	2	2
3	1	−1	0	0	0	0	1	1
4	0	0	1	0	−1	0	1	1
5	0	0	0	1	0	−1	1	1

Proceeding analogously with the remaining sets, the respective coordinates would be as follows:

-
$I^{\left(3\right)}=(\ln\frac{{\left(x_{1}x_{3}x_{4}x_{5}\right)}^{1/4}}{{\left(x_{2}x_{6}\right)}^{1/2}},\ln\frac{{\left(x_{1}x_{3}\right)}^{1/2}}{{\left(x_{4}x_{5}\right)}^{1/2}},\ln\frac{x_{1}}{x_{3}},\ln\frac{x_{4}}{x_{5}},\ln\frac{x_{2}}{x_{6}})$ ,
-
$I^{\left(4\right)}=(\ln\frac{{\left(x_{1}x_{2}x_{3}x_{4}\right)}^{1/4}}{{\left(x_{5}x_{6}\right)}^{1/2}},\ln\frac{{\left(x_{1}x_{4}\right)}^{1/2}}{{\left(x_{2}x_{3}\right)}^{1/2}},\ln\frac{x_{1}}{x_{4}},\ln\frac{x_{2}}{x_{3}},\ln\frac{x_{5}}{x_{6}})$ ,
-
$I^{\left(5\right)}=(\ln\frac{{\left(x_{1}x_{2}x_{4}x_{5}\right)}^{1/4}}{{\left(x_{3}x_{6}\right)}^{1/2}},\ln\frac{{\left(x_{1}x_{5}\right)}^{1/2}}{{\left(x_{2}x_{4}\right)}^{1/2}},\ln\frac{x_{1}}{x_{5}},\ln\frac{x_{2}}{x_{4}},\ln\frac{x_{3}}{x_{6}})$ ,
-
$I^{\left(6\right)}=(\ln\frac{{\left(x_{1}x_{2}x_{5}x_{6}\right)}^{1/4}}{{\left(x_{3}x_{4}\right)}^{1/2}},\ln\frac{{\left(x_{1}x_{6}\right)}^{1/2}}{{\left(x_{2}x_{5}\right)}^{1/2}},\ln\frac{x_{1}}{x_{6}},\ln\frac{x_{2}}{x_{5}},\ln\frac{x_{3}}{x_{4}})$ .

**Figure 1.**
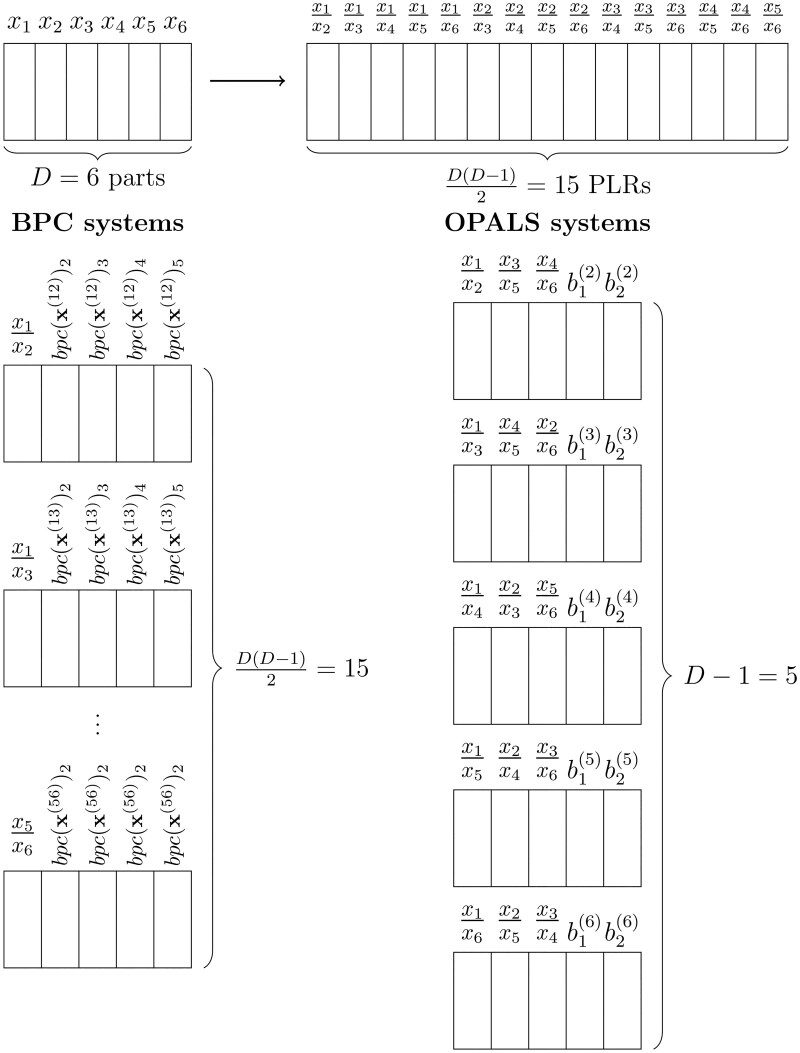
Schematic comparison of the bpc and OPALS approaches to obtain orthonormal pairwise logratios from a 6-part composition. At the top, the step from D=6 parts to D(D−1)/2 pairwise logratios is illustrated. Then, the orthonormal coordinate systems required by each approach to cover all those pairwise logratios are represented (15 in the bpc case and 5 in the OPALS case).

The optimal procedure embedded into the OPALS algorithm to produce the final D−1 coordinate systems required to include all PLRs effectively for further analysis, instead of the D(D−1)/2 systems resulting from the bpc approach, is sketched in Fig. 1. However, it is relevant to note that the total number of resulting features, the PLRs in our case, remains quadratic. In high-dimensional scenarios, constructing and modelling all PLRs can still be challenging. In such cases, it may be useful to first explore the empirical distribution of PLRs containing a given part, e.g. through kernel density estimation (see Sections 4.1 and 4.2). This exploratory step can help to identify extreme values, assess variability, and get insight about the most relevant PLRs.

Although our main focus here is on demonstrating how to efficiently construct and work with the full set of PLRs, we acknowledge that various strategies exist to reduce dimensionality or select informative subsets of features. For instance, the non-overlapping selection procedure proposed by Coenders and Greenacre (2023) allows for targeted selection of relevant PLRs. Moreover, sparse principal component analysis, as introduced in Nesrstová *et al.* (2025), allows retaining key information using a reduced set of features. Such approaches are particularly valuable when the aim is, from the start, to constrain the analysis to a smaller number of parts or logratios, rather than considering the entire data set.

### 3.3 Link between ordinary and backward pivot coordinates in regression analysis

Both balance (5) and pivot coordinates (2) have been commonly used in ordinary regression analysis with the composition playing an explanatory role (Hron *et al.* 2012, McGregor *et al.* 2020, Coenders and Pawlowsky-Glahn 2020). However, new challenges are posed by high-dimensional compositions, both computational and interpretational. This has motivated new developments, particularly in relation to high-throughout and microbiome data analysis (Gloor *et al.* 2017). For example, a number of algorithms seeking to identify optimal predictive balances have been proposed (Rivera-Pinto et al. 2018, Quinn and Erb 2020, Gordon-Rodriguez *et al.* 2021, Nesrstová *et al.* 2023b, Saperas-Riera *et al.* 2023), penalized regression based on clr coefficients and logcontrast regression models are discussed in Susin *et al.* (2020). Note that using clr coefficients, or ordinary pivot coordinates (Kalivodová *et al.* 2015), in this context involves the computation of geometric means of large collections of compositional parts, which can cause numerical issues and hamper the assessment of their actual influence on the response variable. Recent extensions of the pivot coordinate approach optimized for the case of high dimensions are presented in Štefelová *et al.* (2021, 2023). Moreover, some works focusing on PLRs include Bates and Tibshirani (2019) and Coenders and Greenacre (2023).

Sticking to the olr coordinate framework, orthonormal PLRs through the bpc method (6) have been recently embedded into regression modelling (Hron *et al.* 2021, Nesrstová *et al.* 2023a). The OPALS algorithm introduced in Section 3.2 facilitates extending this to the high-dimensional regression case. Unlike ordinary pivot coordinates, which are actually aggregating information from PLRs into single coordinates as noted in Section 2, stepping down to the PLRs level should allow for a more detailed account of the compositional parts being relevant in regression analysis. This, in turn, should contribute to improving tasks of practical interest such as biomarker identification in biological applications. Interestingly, a link can be established between regression on ordinary and pivot bpcs, which further reinforces the relationship between the aggregated and fine-grain approaches. Thus, considering a regression model with real response *y* formulated in terms of ordinary pivot coordinates (with the *l*th compositional part taking the leading role, l=1,…,D) given by


(11) $$y_{i}=\beta_{0}+\beta_{1}^{\left[l\right]}z_{i1}^{\left[l\right]}+\cdots +\beta_{D-1}z_{i,D-1}^{\left[l\right]}+\varepsilon ,$$

and the model formulated in terms of bpcs as


(12) $$y=\beta_{0}+\beta_{1}^{\left(lr\right)}\text{bpc}{\left(x^{\left(lr\right)}\right)}_{i1}+\cdots +\beta_{D-1}^{\left(lr\right)}\text{bpc}{\left(x^{\left(lr\right)}\right)}_{i,D-1}+\varepsilon ,$$

for any l,r=1,…,D (l≠r) and the index i=1,…,n referring to the observations, it holds that


(13) $$\beta_{1}^{\left[l\right]}=\frac{2}{D}\left[\beta_{1}^{\left(l1\right)}+\cdots +\beta_{1}^{\left(l,l-1\right)}+\beta_{1}^{\left(l,l+1\right)}+\beta_{1}^{\left(lD\right)}\right],$$

which nicely reflects the construction of the first pivot coordinate (3).

For the regular case where n>D, this relationship results from the basic properties of orthogonal (orthonormal) coordinate systems and regression coefficients estimated by the usual least squares method. However, when n<D, as it is often the case with data derived from high-throughput technologies, it is well-known that least squares estimation is not feasible. A popular alternative is using Partial Least Squares (PLS) estimation, which deals with the estimation problem by projection into a low-dimensional space of uncorrelated latent variables (PLS components or factors); see e.g. Varmuza and Filzmoser (2009). The model parameters are determined by maximizing the covariance between latent variable scores and the response variable. An increasing number of PLS components tends to improve model performance until an optimal number of components is reached, which is typically determined by cross-validation. The PLS method working in combination with adapted versions of the pivot coordinate approach has been recently introduced for regression and classification purposes in Kalivodová *et al.* (2015) and Štefelová *et al.* (2023, 2021).

**Figure 2.**
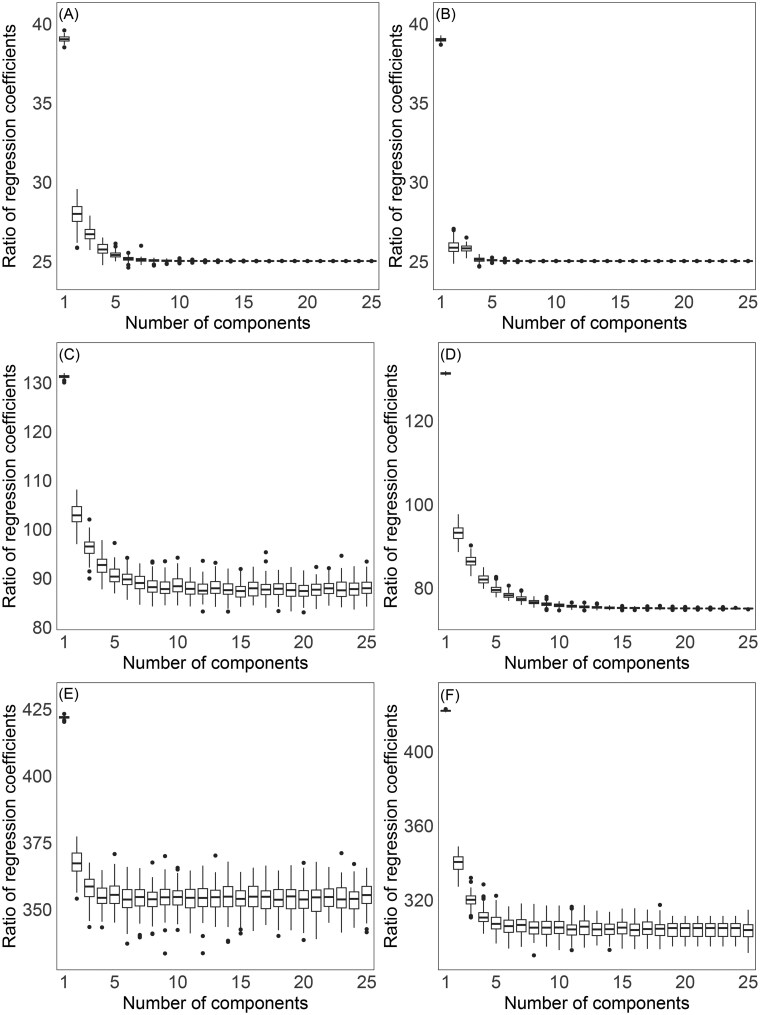
Ratios between coefficients PLS regression models based on ordinary and backwards pivot coordinates for various numbers of observations and compositional parts, with increasing numbers of PLS components. (A) Scenario 100 × 50, (B) Scenario 200 × 50, (C) Scenario 100 × 150, (D) Scenario 200 × 150, (E) Scenario 100 × 450, (F) Scenario 200 × 450.

It is then of interest to look at how the relationship (13) translates into the high-dimensional (n<D) case. This is done here through a small simulation study. For a series of combinations of the number of observations and the number of compositional parts, given by n={100,200} and D={50,150,450}, respectively, compositional data were simulated from a normal distribution on the simplex (Pawlowsky-Glahn *et al.* 2015). This corresponds to an ordinary multivariate normal distribution in olr coordinates and, hence, in pivot coordinates (2) as a particular case. Thus, random values of a response variable *y* were generated by regression on pivot coordinates (normally distributed and setting zero means, unit variances, and uniform covariances equal to 0.7) as


(14) $$y_{i}=\beta_{0}+\beta_{1}z_{1}+\cdots +\beta_{D-1}z_{D-1}+\varepsilon_{i},\;i=1,\ldots ,n,$$

where the regression coefficients were set to $\beta_{0}=0$ and $\beta_{1}=\cdots =\beta_{D-1}=1$, with $\varepsilon_{i}∼N(0,1)$. PLS regression was fitted for each pivot coordinate system and the ratios between the sum of regression coefficients from the right-hand side of (13) and $\beta_{1}^{\left[l\right]}$ were computed for increasing numbers of PLS components (based on 100 simulation runs). Figure 2 summarizes the results, where it can be observed that the ratio stabilizes by D/2 in all regular cases (n>D) as expected. The ratio is no longer constant for the case n<D; however, it is fairly stable from a reasonable number of PLS components. Consequently, these results support the fact that the regression coefficient of the first pivot coordinate $z_{1}^{\left[l\right]}$ represents the aggregation (up to a constant factor) of regression coefficients corresponding to $\text{bpc}{\left(x^{\left(l1\right)}\right)}_{1},\ldots ,\text{bpc}{\left(x^{\left(l,l-1\right)}\right)}_{1},\text{bpc}{\left(x^{\left(l,l+1\right)}\right)}_{1},\ldots ,\text{bpc}{\left(x^{\left(l,D\right)}\right)}_{1}$ and, hence, to the respective PLRs. Thus, it can be understood that such orthonormal PLRs are decomposing the information carried out by the respective pivot coordinates in regression analysis (and analogously if clr coefficients were used instead).

## 4 Illustrative examples

The use of the OPALS algorithm introduced above is demonstrated here by means of two examples from molecular biology. The first one refers to a regression analysis aiming to investigate the association between metabolite signals measured in the rumen of cattle and their greenhouse gas emissions. Although the data set is not strictly high-dimensional, in the sense that it is not a wide data set where n<D, it includes a large number of signals. Thus, a form of regularized regression such as PLS regression is convenient to deal with high correlations between them as commonly found with this type of high-throughput data. The second example specifically involves wide data, and refers to gut microbiome data analysed with the purpose of identifying biomarkers able to differentiate liver cirrhosis patients from healthy controls.

### 4.1 Regression analysis of metabolomic profiles

In Bica *et al.* (2020), nuclear magnetic resonance (NMR) was used to produce spectral data representing metabolomic profiles generated during the fermentation of food in the rumen of cattle. The raw samples went through a number of ordinary preprocessing stages, with final measurements corresponding to normalized integrals of the area under the signal peaks. These metabolomic profiles are known to be associated with livestock methane yields, which are in turn linked to global warming. There is then an interest in identifying associations between metabolites and methane emission (measured as CH_4_ in grams per kilogram of dry matter intake using respiration chambers). This information is e.g. relevant to design animal diets which may help to reduce greenhouse gas emissions from livestock.

The data set consists of n=211 rumen fluid samples, with D=126 integrals per sample. A predictive model of methane emissions was built using PLS regression Wold *et al.* (2001) applied to (12), where all possible PLRs between metabolites were considered as predictors and the response variable (methane emission) was log-transformed to accommodate its relative scale. Both predictors and response variable were centred, so that the intercept $\hat{\beta_{0}}$ is excluded from the model. The number of PLS components was set to 2 based on minimizing the root-mean-square error of prediction (RMSEP) through 10-fold cross-validation and choosing the simplest model amongst whose within one standard deviation of the minimum (so-called one standard error rule). A total of 125 regression models were generated, one for each orthonormal coordinate system, enabling the selection of all possible 7875 PLRs using the OPALS algorithm (Section 3.2). The resulting regression coefficient estimates were standardized dividing by the standard deviation from B=1000 bootstrap resamples. Statistical significance at the usual 5% level was determined using the 2.5% and 97.5% quantiles of the standard normal distribution as cut-off values.

Table 6 summarizes the results, where the top 50 signals are sorted according to the total number of statistically significant PLS regression coefficients of PLRs they are involved in. Note that some correspond to known metabolites and thus their actual name is used, whereas the rest are simply labelled by an ‘I’ (integral) followed by a number indicating position in the metabolomic profile. The ‘Total’ column indicates that total number of significant coefficients, while the ‘Positive’ and ‘Negative’ columns splits this according to the sign or direction of the relationship, either positive or negative, respectively. For instance, PropionateCH3.1 at the very top is involved in 124 significant regression coefficients (of PLRs, where PropionateCH3.1 was in the numerator of each ratio), with all of them having negative sign. Therefore, the methane yield is expected to decrease on average with an increasing dominance of PropionateCH3.1 relative to the metabolites in the denominator of the corresponding PLRs. Prominent biomarkers previously associated with methane yield, such as the volatile fatty acids in the NMR profile including species of propionate (PropCH3.1, PropCH3.2), butyrate (ButCH2b.1, ButCH3.1), and acetate (Palarea-Albaladejo *et al.* 2017), are also highlighted in the current analysis; and the direction of the associations is equally coincident with previous studies. Moreover, signals known to be linked to glucose (I72-73, I80-82, I87), amino acids (I131), hypoxanthine (I11), uracil (I17, I47), and tyrosine (I24) are found on the top list.

**Table 6.** Top 50 nuclear magnetic resonance ruminal metabolite signals associated with livestock methane emissions, ranked by the number of significant PLS regression coefficients in which they are involved across orthonormal pairwise logratio coordinates.**^a^**

Metabolite	Total	Positive	Negative	Metabolite	Total	Positive	Negative
**PropCH3.1**	124	0	124	**I2**	85	3	82
*I54*	123	123	0	*I35*	85	82	3
**I50**	120	0	120	**I82**	85	3	82
**I116**	119	1	118	**I128**	84	6	78
*I88*	116	116	0	I8	82	77	5
**I17**	115	2	113	I5	81	79	2
**I47**	112	1	111	**I103**	80	7	73
*I34*	110	109	1	**I68**	79	6	73
**I89**	110	1	109	I9	78	76	2
*I0*	107	107	0	*I40*	76	71	5
*I43*	107	106	1	*I25*	75	64	11
*I36*	105	104	1	*I111*	75	64	11
*ButCH2b.1*	105	104	1	**I22**	74	65	9
*ButCH3.1*	104	103	1	*I24*	74	66	8
*I6*	99	97	2	I49	74	73	1
**I87**	99	3	96	*I119*	74	62	12
**I99**	99	1	98	I16	73	62	11
**PropCH3.2**	96	4	92	I64	73	5	68
**I11**	95	5	90	*I18*	72	63	9
**I73**	93	6	87	*I32*	72	62	10
I96	92	90	2	*I37*	72	64	8
**I80**	89	4	85	I118	72	57	15
**I131**	87	6	81	*I29*	71	61	10
**I81**	86	3	83	**I72**	71	8	63
*Acetate*	86	81	5	I106	71	18	53

a Columns show the total count of significant coefficients and the distribution of positive and negative signs. Metabolite labels shown in *italics* and **bold **refer to those also identified in previous analysis using weighted pivot coordinates, with *italics* indicating a positive direction and **bold** indicating a negative direction of the association. Labels without formatting correspond to metabolites that were not identified in the previous analysis.

This same data set was used in Štefelová *et al.* (2021) to illustrate marker discovery within a compositional framework based on weighted pivot coordinates, which are aimed at downplaying the influence of noisy signals in the aggregating first coordinate used in the ordinary pivot coordinates approach (Section 2). For comparison with this latter, biomarkers identified in Štefelová *et al.* (2021) are shown in *italics* and **bold** in Table 6, indicating positive and negative associations with the response variable, respectively. When looking at the dominant sign in each case, the results show overall agreement. The great majority of signals amongst these top 50 coincide between both approaches, with some additional signals highlighted by the current orthonormal PLR approach via OPALS algorithm. This is expectable, given that the purpose of this latter is precisely identifying the most elemental sources of association.

**Figure 3.**
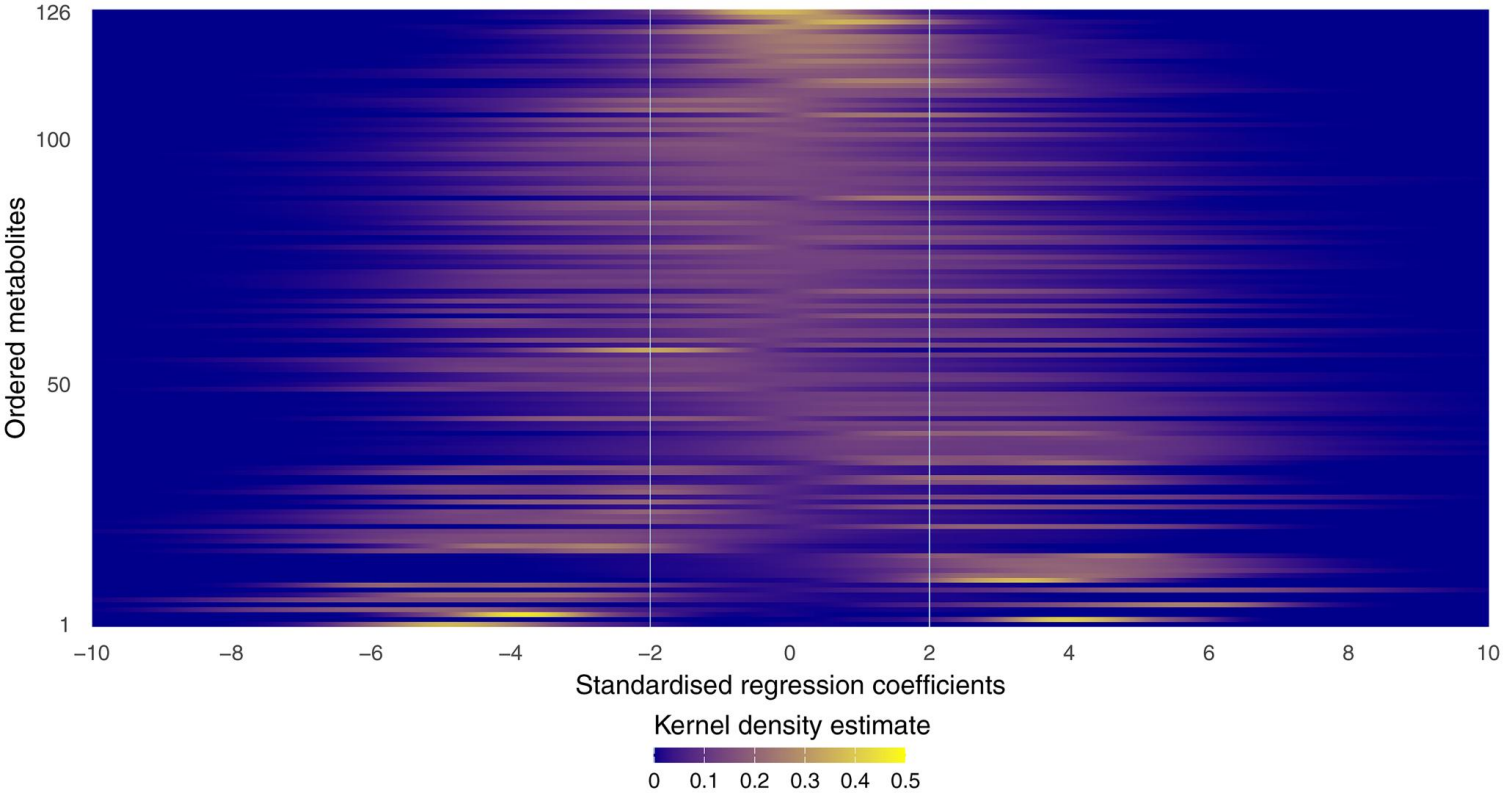
Heatmap of the empirical distributions of standardized PLS regression coefficient estimates associated to each metabolite, ordered according to number of significant coefficients. The vertical lines indicate 2.5% and 97.5% quantile cut-off limits used to determine statistical significance at the 5% level.

The results for all the metabolites are depicted in Fig. 3, where the empirical distributions of standardized regression coefficients corresponding to PLRs linked to each one of them are estimated using kernel density smoothing. As for Table 6, the metabolites have been ordered along the y-axis according to the number of statistically significant coefficients they are involved in (in increasing number from top to bottom). The vertical lines represent the 2.5% and 97.5% quantile cut-off limits used for statistical significance as described above. Thus, a concentration of regression coefficients within the limits (reflected by a higher intensity of the yellow shade in that region of the heatmap) corresponds to non-significance and hence, to less relevance of the metabolite in the association with methane emission. For example, the bottom row corresponds to PropCH3.1, with all significant negative coefficients in agreement with Table 6. Moreover, the top of the heatmap corresponds to the least influential metabolites, with most associated PLS regression coefficients falling in between the significance limits.

**Figure 4.**
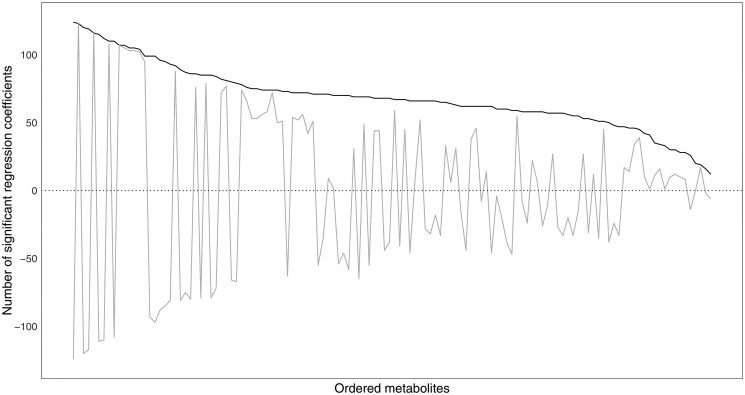
Total number of significant standardized PLS regression coefficients associated to each metabolite (black line) and difference between number of positive and negative ones amongst them (grey line). Metabolites represented on the x-axis in decreasing order according to total number of significant coefficients associated to them.

It is also interesting to look at the distribution of the signs of the PLS regression coefficients for the collection of significant PLRs associated to each metabolite. Figure 4 presents the total number of significant coefficients (black line) along with the difference between the number of positive and negative ones amongst them (grey line). The corresponding metabolites are ordered according to such total number on the x-axis (i.e. in the same order as for Table 6 from left to right). It can be clearly observed that the most influential metabolites are linked to coefficients very much agreeing on the sign, being nearly all either positive or negative. Contrarily, higher heterogeneity in signs tends to occur as the relevance of the metabolites decays (note the differences approaching zero).

### 4.2 Differential expression analysis from microbiome data

This example involves a landmark shotgun microbiome dataset corresponding to stool samples from 216 Chinese individuals, consisting of 114 liver cirrhosis patients and 102 healthy controls (Qin *et al.* 2014, Champion *et al.* 2023). Liver cirrhosis results from a number of chronic liver diseases. It shows a very specific pattern in the gut microbiome, including the invasion of oral species in the gut [called the ‘oral-gut-liver’ axis in the literature (Acharya *et al.* 2017)]. Abundances of so-called metagenomic species pan-genomes (MSPs) (Plaza Oñate *et al.* 2019, 2021) were computed as the mean abundance of 100 marker genes selected for each one of them, with these consisting of counts normalized by gene length to account for sequencing depth. This resulted in a collection of 1990 MSPs from 216 individuals. However, because microbiome data commonly contain many zeros, we included only MSPs with at least 20 non-zero observations out of 216 for a given MSP (around 10%). This filtering step then led to the final 216×456 abundance data set used for analysis.

The purpose was to identify meaningful MSPs distinguishing between both treatment groups, and this was set up as a binary classification problem tackled here by PLS discriminant analysis (PLS-DA) modelling (Barker and Rayens 2003). We followed the same strategy as in the previous example, including analogous calculation of bootstrap-based significance tests for the model coefficients. The optimal number of PLS components was determined here by maximizing classification accuracy into diseased and healthy treatment groups, resulting in six PLS components. However, only the first three were eventually used after applying the one-standard-error rule, which helps to simplify and prevents from excessive noise in the model. In fact, the classification errors using either three or six PLS components were very much comparable (0.1111 against 0.1018, respectively, based on leave-one-out cross-validation). For reference, we conducted an ordinary differential abundance analysis based on multiple statistical testing as often conducted in the area [see e.g. Solé *et al.* (2021), Berland *et al.* (2023), Thirion *et al.* (2023)]. Thus, Wilcoxon’s tests were applied individually across MSPs aiming to identify those involved in the differentiation between the two treatment groups. This included the use of the Benjamini-Hochberg’s adjustment to control for false discovery rate in multiple testing (Benjamini and Hochberg 1995). Table 7 provides the ordered list of top 50 MSPs identified by the PLS-DA method based orthonormal PLRs obtained by the OPALS algorithm. Moreover, similarly to Table 6, the counts of total number of statistically significant PLS-DA model coefficients of orthonormal PLR coordinates associated with each MSP are reported, along with their allocation to a treatment group according to the leading abundance. MSP labels shown in **bold** indicate those that were detected by the customized PLS-DA modelling but not by the ordinary Wilcoxon’s test approach. It can then be observed that both methods mostly agreed in their findings, although a few biomarkers were only detected by the former. In any case, it is important to note that the setting of the two methods is notably different: whereas the PLS-DA based on orthonormal PLRs considers both the multivariate and compositional nature of the data, Wilcoxon’s testing works univariately and ignores the potential interdependences between MSP abundances.

**Table 7.** Top 50 metagenomic species (MSPs), ranked by the number of significant PLS regression coefficients in which they are involved across orthonormal pairwise logratio coordinates, along with treatment group association based on leading abundance (diseased or control group).^a^

MSP	Total	Diseased	Control	MSP	Total	Diseased	Control
msp_0313	453	453	0	msp_0095	323	0	323
msp_0881	440	440	0	msp_0884	319	315	4
msp_0077	432	432	0	msp_0043	318	0	318
msp_0148c	427	426	1	msp_0988	314	0	314
msp_0086	426	425	1	msp_0024	313	308	5
msp_0227	407	0	407	msp_0090	312	311	1
msp_0712	407	406	1	msp_1325	311	0	311
msp_0380	397	396	1	msp_0490	306	0	306
msp_0422	394	0	394	msp_0163	303	0	303
msp_0215	392	0	392	msp_0777	299	0	299
msp_0055	382	381	1	msp_0364	298	293	5
msp_0591	381	0	381	msp_0058	297	294	3
**msp_0023**	377	376	1	**msp_0134**	293	0	293
msp_0288	374	373	1	**msp_0510**	288	283	5
msp_0056	369	366	3	msp_0346	287	0	287
msp_0013	363	362	1	msp_0844c	287	2	285
msp_0075	363	362	1	msp_0977	287	1	286
msp_0742	360	357	3	msp_0898	282	0	282
msp_0224	359	358	1	msp_0045	281	280	1
**msp_0290**	359	357	2	**msp_0025**	280	278	2
msp_0186	358	0	358	msp_0036	280	0	280
msp_0063	347	0	347	msp_0044	279	272	7
msp_0015	336	0	336	msp_1599	279	272	7
**msp_0098**	324	319	5	**msp_0388**	273	271	2
msp_0780	324	0	324	msp_0244	270	263	7

a MSP labels shown in **bold** indicate those not detected by the ordinary Wilcoxon’s test approach.

Moreover, we use this example in the high-dimensional case to point out the computational efficiency of the proposed OPALS algorithm in comparison with the default bpc calculations based on D(D−1)/2 coordinate systems as sketched in Section 2. For this, subsets of MSPs were incrementally selected from the original collection in the microbiome data set, starting with the first D=50 and then augmenting them in batches of 50 up to D=450 (all the n=216 samples were used). All non-overlapping PLRs were selected and the PLS-DA model was fitted following each procedure. Three PLS components were considered and the corresponding model coefficients were obtained. Table 8 summarizes the total computing times in minutes required in each case. This was measured on a desktop computer equipped with a 2.71 GHz 11th Gen Intel Core i5 processor and 16 GB of RAM.

**Table 8.** Comparison of computing times (in minutes) to obtain all required orthonormal pairwise logratios between the proposed OPALS algorithm, default backward pivot coordinate (bpc) calculations based on D(D−1)/2 systems, for incremental numbers of components *D* taken from the original microbiome data set.^a^

	*D*								
Method	50	100	150	200	250	300	350	400	450
OPALS algorithm	0.09	0.67	2.3	5.3	12.8	19.2	27.7	42	61
Default bpc approach	0.12	0.8	4.2	12.4	51.9	–	–	–	–
STEPR method	0.35	5.54	7.9	13.4	19.5	28.7	35.3	47	65.5

a STEPR method for searching non-overlapping pairwise logratios included for further comparison.

It can be observed that the computing time elapsed for the smallest values of *D* is fairly comparable; however, the gap increases significantly from about D=200, with calculations using the default procedure turning unfeasible from D=300 on. Thus, for example, the computation stopped in memory allocation failure after ∼2 hours running in the D=300 case, being still in the process of arranging all the coordinate systems. However, the OPALS algorithm completed the entire task including model fit in about 19 minutes. For the largest D=450 case considered, the OPALS algorithm successfully prepared the coordinate systems containing all the 101 025 PLRs and PLS-DA model fitting was conducted within 61 minutes. Additionally, we included the STEPR procedure proposed by Coenders and Greenacre (2023) in the comparison, which is also designed to work with PLRs as predictors. Unlike OPALS, which constructs a complete and orthogonal representation of all PLRs for subsequent analysis, STEPR focuses on directly selecting a subset of non-overlapping PLRs that are most relevant according to predefined criteria. Although we found their computing times to be fairly comparable, with OPALS performing slightly better, the two approaches serve different purposes. That is, either working with the entire structure of relative information to be further analysed within an orthonormal logratio framework, in the case of OPALS, or identifying a compact subset of predictive features, in the case of STEPR.

**Figure 5.**
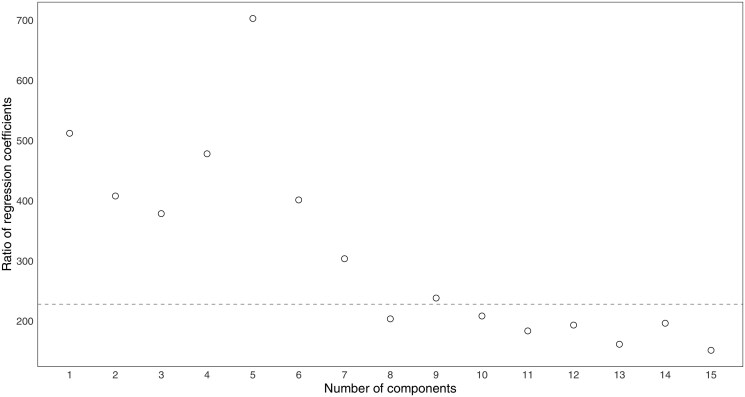
Ratios between PLS-DA model coefficients based on ordinary and backward pivot coordinates for increasing numbers of PLS components from the liver cirrhosis microbiome data example. The horizontal dashed line indicates the *D*/2 threshold.

Lastly, we used this high-dimensional data set to illustrate empirically the relationship between the coefficients of the models based on ordinary and bpcs, or generally any orthonormal logratio coordinate representation involving PLRs, as discussed in Section 3.3. Namely, analogously to Figs 2F and 5 shows the ratios between coefficient from the former and the sum of coefficients from the latter for the first 15 PLS components, averaged across all compositional parts. It can be observed that such ratio stabilizes around a number smaller than D/2 from eight PLS components on, thus confirming the pattern seen previously by simulation.

## 5 Conclusion

Pairwise logratios (PLRs) contain the most elemental information in compositional data. In some contexts, it might be preferable working with them directly instead of relying on one of the common forms of aggregated logratio coordinates used in the literature. PLRs are more directly linked to the original compositional parts, which generally facilitates context-specific interpretability. The concept of orthonormal logratio coordinates, namely backward pivot coordinates (bpcs), provides a well-founded and consistent approach to conduct compositional data analyses based on PLRs, where invariance of statistical models to rotations and/or shifts of the data points is required. Thus, for instance in regression analysis, they guaranteed that the regression coefficient associated to a certain PLR is the same regardless of the particular coordinate system in which it is included. However, as discussed above, implementing this approach in high dimensions rapidly leads to an unbearable computational burden.

This work introduces an efficient procedure, the OPALS algorithm, to deal with such computational challenge. It allows obtaining all orthonormal PLRs by solving a combinatorial problem in the form of a Latin square. This leads to a total of D−1 coordinate systems each containing D/2 PLRs, so that the computation burden is notably reduced when compared to the default bpc calculations based on D(D−1)/2 systems. Its use was illustrated above in the context of ordinary regression and classification analysis, including models based on the partial least squares method designed for high-dimensional data sets. But it could be equally combined with any robust counterparts, such as models based on least trimmed squares or MM estimators, or other regression methods for high dimensions such as principal component regression.

From a practical perspective, the main advantage of the OPALS algorithm is that it allows to work with the complete collection of orthonormal PLRs in a computationally efficient manner. This ensures that no potentially relevant pairwise relationships are omitted, unlike in the case of applying some automatic variable selection or sparse approach directly. This can be particularly valuable in exploratory analysis, analyses focused on interpretability, or as a starting point for subsequent feature selection. A potential drawback, however, is that the number of resulting PLRs still grows quadratically with the number of compositional parts. Although OPALS reduces the number of orthonormal coordinate systems required dramatically, it does not aim to reduce the number of features themselves. Therefore, in particularly demanding high-dimensional settings, further steps such as filtering, ranking, or applying some sparsity-inducing or regularization methods may be useful to manage such volume of information.

Ultimately, the proposed method greatly alleviates the computation burden associated with compositional data analysis based on orthonormal PLRs, enabling further scalability to deal with high-dimensional data as commonly generated in modern fields such as the omics sciences. By efficiently handling PLRs within an orthonormal coordinate approach, the method contributes to enhance variable selection and stability of statistical models; leveraging the fine-grain information contained in PLRs to e.g. refine biomarker discovery and, more generally, untapping new opportunities for the application of the logratio methodology in these areas.

## Supplementary Material

vbaf229_Supplementary_Data

## Data Availability

Differential expression analysis from microbiome data are available here: https://entrepot.recherche.data.gouv.fr/dataset.xhtml?persistentId=doi:10.15454/FLANUP.
